# Does Maternal Country of Birth Matter for Understanding Offspring’s Birthweight? A Multilevel Analysis of Individual Heterogeneity in Sweden

**DOI:** 10.1371/journal.pone.0129362

**Published:** 2015-05-28

**Authors:** Shai Mulinari, Sol Pia Juárez, Philippe Wagner, Juan Merlo

**Affiliations:** 1 Department of Sociology, Faculty of Social Sciences, Lund University, Lund, Sweden; 2 Unit of Social Epidemiology, Faculty of Medicine, Lund University, Malmö, Sweden; 3 Centre for Health Equity Studies, Stockholm University and Karolinska Institutet, Stockholm, Sweden; Yokohama City University, JAPAN

## Abstract

**Background:**

Many public health and epidemiological studies have found differences between populations (e.g. maternal countries of birth) in average values of a health indicator (e.g. mean offspring birthweight). However, the approach based solely on population-level averages compromises our understanding of variability in individuals’ health around the averages. If this variability is high, the exclusive study of averages may give misleading information. This idea is relevant when investigating country of birth differences in health.

**Methods and Results:**

To exemplify this concept, we use information from the Swedish Medical Birth Register (2002–2010) and apply multilevel regression analysis of birthweight, with babies (n = 811,329) at the first, mothers (n = 571,876) at the second, and maternal countries of birth (n = 109) at the third level. We disentangle offspring, maternal and maternal country of birth components of the total offspring heterogeneity in birthweight for babies born within the normal timespan (37–42 weeks). We found that of such birthweight variation about 50% was at the baby level, 47% at the maternal level and only 3% at the maternal countries of birth level.

**Conclusion:**

In spite of seemingly large differences in average birthweight among maternal countries of birth (range 3290–3677g), knowledge of the maternal country of birth does not provide accurate information for ascertaining individual offspring birthweight because of the high inter-offspring heterogeneity around country averages. Our study exemplifies the need for a better understanding of individual health diversity for which group averages may provide insufficient and even misleading information. The analytical approach we outline is therefore relevant to investigations of country of birth (and ethnic) differences in health in general.

## Introduction

Offspring’s birthweight is a rough but frequently studied reproductive outcome that is related to both maternal and offspring health status [1]. Low birthweight can be a consequence of intrauterine growth restriction, which leads to babies being small for gestational age (SGA). In turn, being SGA appears to increase the average risk of neonatal mortality and morbidities [2] as well as of major medical problems across the life course [3–13]. Therefore, the identification of factors that condition birthweight raises interest in public health and preventive medicine [14–15]. Notably, information on birthweight is relatively easy to obtain and is routinely used by the World Health Organization for performing epidemiological comparisons between countries [16].

Offspring birthweight also seems conditioned by the social and economic circumstances of the mother, and it may vary across and between immigrant and non-immigrant populations residing in a country. A simple overview of the literature indicates that a considerable number of public health studies have investigated nationwide differences in offspring birthweight by maternal country of birth (MCB) (sometimes used as a proxy for ethnicity). The results of those studies seem to be disparate and to depend, for instance, on the chosen MCB and length of stay in the host country, and on whether or not the mother is the first migrant generation [17–31]. The importance of those studies is justified by our interest in identifying health inequities and the consequent demand for public health interventions aimed at eliminating such unwarranted differences. From this perspective, most studies on ethnic differences in health outcomes such as birthweight, including those using MCB as a way to categorise people’s ethnicity [32], make two implicit assumptions.

In the first place, it is assumed that the MCB exerts a *general contextual influence* on each and every one of individuals born in the same country [33–34]. This general influence is expected to be a result of shared conditions, for instance, common social or national experiences, shared cultural heritage, symbolic systems such as religion, and other circumstances like dress style, physical appearance, and so forth. There can also be common experiences of deprivation as well as a specific pattern of risk factors conditioned by particular cultural and lifestyle habits related to the MCB. For some immigrant groups, a general influence of the MCB may also be caused by the experiences of migration, as well as discrimination and socioeconomic disadvantage suffered in the host country [35–40].

In the second place, it is assumed that the *general contextual influence* described above can be analysed by quantifying between-countries differences in average health outcomes. For this purpose, measures of association like beta coefficients for continuous variables (e.g. birthweight) or odds ratios for dichotomous variables (e.g. Low Birthweight: <2,500 g) are traditionally used. Frequently, a particular country (often the host country) is chosen as reference in the comparisons. On occasions, countries are classified by coarse categorisations based on geographical or economic criteria. Scholars may also create ‘league tables’ by ranking countries according to their average values (e.g. mean birthweight) but without any specific country as reference.

However, we maintain that most studies performed so far have not been able to appropriately quantify the *general contextual influence* of the MCB on offspring birthweight just because they are exclusively based on population average information [33,41–42]. This situation compromises the understanding of individual health heterogeneity around the averages. In addition, the existence of differences in average values between the host country and the MCB of some immigrants promotes the idea of considering country of birth, and hence, certain ethnicities, as a risk factor for disease [43–44]. From this viewpoint, the evidence of similar or even better average fitness in some immigrant groups compared to natives is interpreted as a ‘healthy migrant effect’ or an ‘immigrant health paradox’ [45–49]. However, if the individual heterogeneity around the country averages is large and health outcome distributions display major overlap between countries, immigrants’ health risks cannot properly be distinguished from natives’, so understanding immigrants’ country of birth as an unhealthy ‘risk factor’ or as a ‘health paradox’ may be unfounded. Indeed, more generally, if the individual heterogeneity in health outcomes around the country averages is large, MCB should not be considered as an appropriate construct for forecasting individual health.

In summary, a possible *general contextual influence* of ethnicity or of MCB on individual health indicators like offspring birthweight is not properly operationalized by measuring differences between group averages when diversity is paramount. Rather, we need a multilevel analytical approach that focuses on analysing both differences between country averages and individual heterogeneity within populations. A possible influence of the MCB is better quantified by measuring the share of the total inter-individual heterogeneity in the health indicator that appears at the country of birth level [50]. A suitable measure for this purpose is, for instance, the variance partition coefficient (VPC) [42,50–52] obtained from multilevel regression analyses. When comparing countries, if the VPC is low (i.e. if the share of the total individual variance in birthweight that can be located at the level of the MCB is low), the actual differences in average values between countries become less relevant, even if these differences are statistically ‘significant’.

In the present study, following an analytical approach previously described [33–34,53–54], we applied multilevel linear regression techniques [41–42,50,55] with babies nested within mothers that in turn were nested within their countries of birth, to investigate differences in birthweight between babies born in Sweden between 2002 and 2010. Using this approach, we disentangled the shares of those offspring differences that were at the levels of the mother and the MCB, respectively. By doing so, we aimed to illustrate the relevance of considering individual-level heterogeneity around group averages when interpreting the general contextual influence of ethnicity or of country of birth on individual health indicators. We analysed a large database including 811,329 babies born in Sweden from 571,876 mothers representing 109 different countries of origin that matched our inclusion criteria.

While we perform a formal empirical analysis of birthweight and our results are restricted to that outcome, our study also has an underlying intention of exemplifying the use of multilevel analysis for the investigation of individual heterogeneity [41]. In this sense, the analytical approach we outline in the present birthweight example is, we believe, of general relevance to epidemiological investigations of country of birth and ethnic differences in health outcomes.

## Methods

### Study population

We used data from all the 938,932 births recorded at the Swedish Medical Birth Register (MBR) between 1 January 2002 and 31 December 2010. The MBR collects detailed and standardised information on nearly all pregnancies in Sweden culminating in delivery [56] and is administered by the National Board of Health and Welfare. Using a unique personal identification number, the MBR was linked to several other registries containing demographic and socioeconomic information and that are maintained by Statistics Sweden. The Swedish authorities prepared the research database and delivered it to us without the personal identification numbers to ensure the anonymity of the subjects. The Regional Ethics Review Board in southern Sweden (DNR 71/2006) approved the construction of the database.

**Fig 1:**
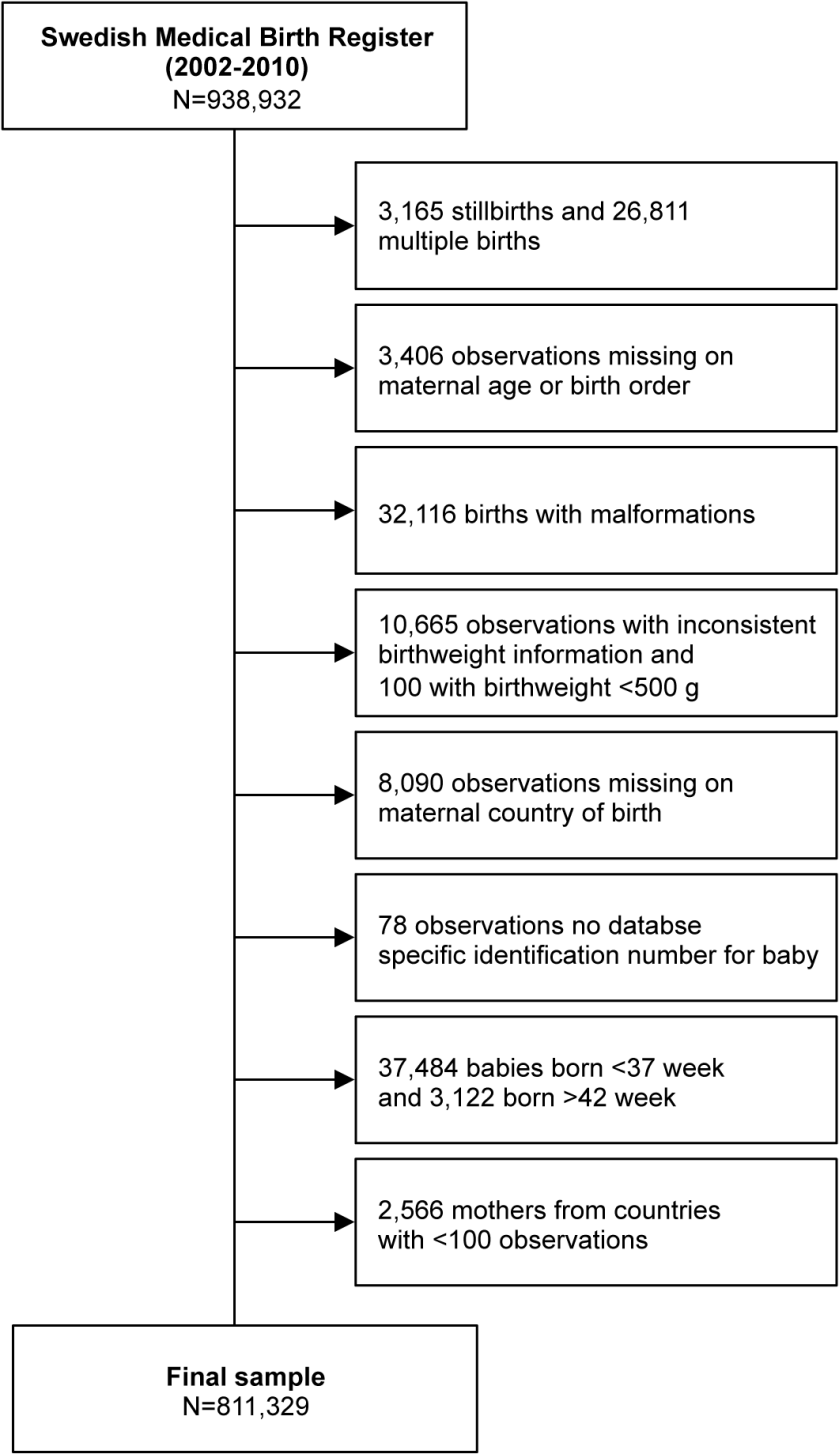
Flow diagram showing the selection of the study population.

The study selection process is shown in Fig 1. We selected singletons born alive (n = 908,956), since it is known that multiple births (n = 26,811) show a different intrauterine growth pattern from gestational weeks 28–30 [57]. We also excluded cases with missing information about maternal age or birth order (n = 3406), babies with malformations (n = 32,116) and babies weighing less than 500 g (n = 100). Following the criteria previously published [58], we excluded babies with inconsistent information on birthweight according to gestational age (n = 10,665). We excluded babies with missing information regarding the MCB (n = 8090) and for whom we did not have any database specific identification number (n = 78). Finally, for the purpose of our study, we excluded babies born preterm (before week 37) (n = 37,484) or post-term (after week 42) (n = 3122), as well as babies from MCB with fewer than 100 observations (n = 2566). The final sample consisted of 811,329 babies born from 571,876 mothers from 109 different countries of origin.

### Assessment of variables

The outcome variable was the birthweight in grams (g). In order to explain possible differences between MCB, we included information on the available maternal and child variables known to be associated with birthweight according to previous publications. Among child characteristics, we considered sex because females are lighter than boys on average [14,59], and we used the males as reference group. We also included gestational age because it is a main predictor of birthweight [60].

Among maternal characteristics, we included information on maternal stature since it is known that short mothers have increased risk of delivering smaller infants [61–62]. We divided this variable into five categories (<150 cm, 150–159 cm, 160–169 cm, 170–179 cm and >179 cm) and a missing category, with mothers with their stature between 160 and 169 cm being the reference category. We also included maternal age at delivery, as extreme ages are strongly associated with lighter babies [63–64]. We categorised this variable into four groups (<20, 20–24, 25–34 and >35 years old), and we considered mothers from the age group 25–34 as the reference category. We included marital status, since it is reported that single mothers have a higher probability of delivering low birthweight babies [65]. We categorised this information into single, widowed or divorced, and married or cohabiting, using the last category as the reference group. We included information on maternal smoking, since this habit is associated with low birthweight [66]. We grouped smoking habits into three categories: non-smoking (reference), moderate (fewer than 9 cigarettes per day), heavy (more than 9 cigarettes per day) and missing information. Information on smoking habits was based on a self-reported questionnaire administered by the midwife at the first antenatal visit (typically between 9 and 12 gestational weeks). Socioeconomic position is related to both maternal and child’s health [67], so we included information on household income. We defined household income as the mean disposable income of parents the year before delivery (the income variable also included parental leave and other benefits). We classified this variable into low, middle and high income (reference) according to tertiles, and we included a missing category when information for any parent was absent. We included dichotomous variables (yes vs no) for maternal hypertension and diabetes, respectively, since these conditions impact on the offspring’s birthweight [68–69].

At the contextual level, we categorised the MCB according to the World Bank (WB) classification of country economies based on the Gross National Income (GNI) per capita using the WB Atlas method [70]. This definition includes four categories (low income, lower-middle income, upper-middle income and high income), and we used high income countries as a reference in the comparisons.

### Statistical analyses

We applied multilevel linear regression to model individual birthweight, with babies at the first level (n = 811,329), mothers at the second level (n = 571,876) and MCB at the third level (n = 109). We performed five consecutive models. The first one only contained a random term for each of the three levels (babies, mothers and MCB) studied. This model simply aimed to describe the components of the total variance in birthweight. The second model included information on maternal stature as a fixed effect since maternal stature is a major determinant of birthweight [61–62]. The third model included also information on the mother’s age since we hypothesised that age differences could account for an important share of the remaining variation. The fourth model included all individual variables as fixed effects. The fifth model extended the previous one by including contextual information about the economic circumstances of the MCB as a fixed effect.

For the estimation of models we first used the restricted generalised least square (RIGLS) method to obtain start values for the final Markov chain Monte Carlo (MCMC) estimations [71–72]. We used the posterior distribution of the parameters obtained by the MCMC method to estimate measures of association (i.e. regression coefficients) and measures of variance as well as their standard error. We use the Bayesian deviance information criterion (BDIC) as a measure of goodness of fit of our models [73]. The idea is that models with smaller BDIC should be preferred to models with larger BDIC.

When it comes to the study of contextual influences (in our case the influence of the MCB on birthweight), the analysis distinguishes between *general* and *specific* contextual influences (also denominated ‘effects’ in observational epidemiology).

#### General contextual influences

We estimated the intercept variance at the baby (σ^2^_b_), the mother (σ^2^_m_) and the MCB (σ^2^_mcb_) levels. Thereafter, we calculated the variance partition coefficients (VPC) as follows,

$${VPC}_{mcb}=(\sigma_{mcb}^{2}/(\sigma_{mcb}^{2}+\sigma_{m}^{2}+\sigma_{b}^{2}))\;*\;100$$ formula 1

$${VPC}_{m}=(\sigma_{m}^{2}/(\sigma_{mcb}^{2}+\sigma_{m}^{2}+\sigma_{b}^{2}))\;*\;100$$ formula 2

$${VPC}_{b}=(\sigma_{b}^{2}/(\sigma_{mcb}^{2}+\sigma_{m}^{2}+\sigma_{b}^{2}))\;*\;100$$ formula 3

where the VPC expresses the share of the total variance (σ^2^_mcb_ + σ^2^_m_ + σ^2^_b_) that is at a specific level.

Given the hierarchical structure of our data, we also measured the intra-class correlation (ICC), which provides information about the correlation in birthweight between two babies randomly chosen from either the same MCB (which corresponds with *formula 1* above) or from the same mother (*formula 4*).

$$ICC_{mcb}=formula\;1$$

$${ICC}_{m}=(\left(\sigma_{mcb}^{2}++\sigma_{b}^{2})/(\sigma_{mcb}^{2}+\sigma_{m}^{2}+\sigma_{b}^{2}\right))\;*\;100$$ formula 4

For every model, we calculated the proportional change in variance (PCV) [55] compared to the ‘empty’ model (i.e. Model 1) for each of the three levels studied to assess the share of the variance explained by subsequent models. The PCV is obtained as follows:

$${ICC}_{m}=(\left(\sigma_{mcb}^{2}++\sigma_{b}^{2})/(\sigma_{mcb}^{2}+\sigma_{m}^{2}+\sigma_{b}^{2}\right))\;*\;100$$ formula 5

#### Specific contextual influences

The specific contextual effects are those appraised by observing differences between MCB in average birthweight. We appraised the specific contextual effects in two ways: by grouping the MCBs according the WB income data and obtaining beta-coefficients, and by plotting the shrunken residuals of the MCB level, which are the differences between the weighted mean birthweight of each country and the average birthweight of all the MCBs.

### Data analysis

We performed the analyses using SPSS 22.0 (IBM corp. USA) and MLwiN 2.31 (Centre for Multilevel Modelling, University of Bristol, Bristol, UK) [74].

## Results

### Characteristics of the population

Table 1 presents the characteristics of the population by the WB classification of economies. Mothers in high-income countries appear to be taller on average, especially in comparison to mothers in lower-middle income countries. It appears that high-income countries have a lower proportion of mothers <25 years, and a higher proportion of families with high or middle incomes than other country income categories. There is also a seemingly higher proportion of missing information on household income in low-, lower middle-, and upper middle-income countries. Lower income countries appear to have fewer smokers and more single mothers.

**Table 1:** Characteristics of the population by maternal country of birth economies according to the World Bank classification based on estimates of gross national income (GNI) per capita.

Income Economies	All Economies		High Income		Upper Middle		Lower Middle		Low Income	
	**N**	%	**N**	%	**N**	%	**N**	%	**N**	%
	811,329	100	688,766	84.89	81,635	10.06	22,788	2.81	18140	2.24
**Number of countries**	109	100	33	30.28	37	33.94	25	22.94	14	12.84
**Mother’s stature**										
<150	2973	0.37	853	0.12	1096	1.34	798	3.50	226	1.25
150–159	94,284	11.62	58,188	8.45	22,099	27.07	9159	40.19	4838	26.67
160–169	41,8305	51.65	357,350	51.88	42,118	51.59	9697	42.55	9140	50.39
170–179	23,6102	29.10	221,169	32.11	11,014	13.49	1629	7.15	2290	12.62
>179	15,850	1.95	15,222	2.21	475	0.58	53	0.23	100	0.55
Missing	43,815	5.40	35,984	5.22	4833	5.92	1452	6.37	1546	8.52
**Mother’s age**										
<20	13,264	1.63	10,426	1.51	2094	2.57	353	1.55	391	2.16
20–24	101,854	12.55	77,520	11.25	17,073	20.91	3937	17.28	3324	18.32
25–34 (ref)	528,979	65.20	456,899	66.36	47,254	57.88	14,308	62.79	10,518	57.98
>34	167,232	20.61	143,921	20.90	15,214	18.64	4190	18.39	3907	21.54
**Sex of baby**										
Male (ref)	414,119	51.04	351,630	51.05	41,578	50.93	11705	51.36	9206	50.07
Female	397,210	48.96	337,136	48.95	40,057	49.07	11083	48.63	8934	49.25
**Household income**										
Highest	267,969	33.03	254,919	37.01	8055	9.87	2497	10.96	1093	6.03
Middle	267,110	32.92	244,605	35.51	15,017	18.40	4601	20.19	2887	15.92
Lowest	267,969	32.86	186,189	27.03	54,384	66.62	14,257	62.56	13,139	72.43
Missing	9686	1.19	3053	0.44	4179	5.12	1433	6.29	1021	5.63
**Maternal marital status**										
Married/cohabiting	731,684	90.18	624,408	90,66	73,631	90.20	20,254	88.88	13,391	73.82
Single	13,567	1.67	9395	1.36	1700	2.08	619	2.72	1853	10.21
Widowed/divorced	28,868	3.56	22,495	3.27	3286	4.03	1002	4.40	2085	11.49
Missing	37,210	4.59	32,468	4.71	3018	3.70	913	4.01	811	4.47
**Smoking at the first prenatal visit**										
Non-smoking	712,291	87.79	602,705	87.51	71,761	87.90	20,8,97	91.70	16,928	93.32
1–9 cig /day	45,261	5.58	38,954	5.66	5246	6.43	739	3.25	322	1.78
>9 cig/day	14,797	1.82	13,067	1.90	1482	1.82	192	0.84	56	0.31
Missing	38,980	4.80	34,040	4.94	3146	3.85	960	4.21	834	4.60
**Hypertension**	29,186	3.60	26,447	3.84	1,689	2.07	571	2.51	479	2.64
**Diabetes**	3,722	0.50	3,068	0.44	378	0.46	112	0.49	164	0.90
**Gestational age (w)**										
Mean (SD)	39.66	(1.27)	39.67	(1.27)	39.54	(1.25)	39.40	(1.26)	39.86	(1.34)
**Birthweight (g)**										
Mean (SD)	3,605	(485)	3,628	(483)	3,488	(468)	3,424	(473)	3,461	(473)

### Measures of association and specific contextual influences

In Table 2, Model 2, we observe that average birthweight increased with the mother’s stature. In Model 3 we observe that mothers younger than 25 years delivered lighter, and mothers older than 34 heavier, babies than women 25–34 years old. Model 4 shows that average birthweight was lower in girls than in boys, and that it increased with gestational age. Compared to non-smoking mothers, light- and heavy-smoking mothers delivered babies that were, respectively, on average 129 g and 179 g lighter. Higher maternal household income associated with lighter children. As expected, maternal hypertension was associated with lighter children and maternal diabetes with heavier children. In Model 5 we show the adjusted average birthweight of babies whose MCB was in the category low, lower-middle or upper-middle income economies, respectively. Such babies were on average 88 g, 52 g and 16 g lighter, respectively, than babies of mothers from high-income countries.

**Table 2:** Multilevel linear regression analysis of babies, mothers and maternal countries of birth, modelling birthweight (in grams)^1^.

	Model 1	Model 2	Model 3	Model 4	Model 5
*Fixed effects parts of models*					
Overall mean (intercept)	3490 [6.2]	3508 [8.2]	3510 [5.1]	3567 [7.4]	3595 [9.9]
**Mother’s stature**					
<150		-232 [8.8]	-231 [9.3]	-185 [8.1]	-184 [8.2]
150–159		-118 [1.9]	-116 [1.9]	-98 [1.7]	-98 [1.7]
160–169		Ref.	Ref.	Ref.	Ref.
170–179		117 [1.4]	115 [1.4]	99 [1.2]	99 [1.2]
>179		242 [4.2]	237 [4.6]	202 [3.7]	202 [3.8]
Missing		5 [2.3]	5 [2.2]	6 [3.0]	6 [3.1]
**Mother’s age**					
<20			-123 [4.1]	-106 [3.7]	-106 [3.7]
20–24			-62 [1.6]	-60 [1.4]	-60 [1.5]
25–34 (ref)			Ref.	Ref.	Ref.
>34			38 [1.3]	51 [1.2]	51 [1.2]
**Sex**					
Male (ref)				Ref.	Ref.
Female				-123 [0.8]	-123 [0.9]
**Gestational age (weeks)**				149 [0.4]	149 [0.4]
**Household income**					
Highest				Ref.	Ref.
Middle				76 [1.1]	76 [1.1]
Lowest				107 [1.2]	107 [1.2]
Missing				12 [4.2]	12 [4.3]
**Maternal marital status**					
Married/cohabiting				Ref.	Ref.
Single				-39 [3.5]	-39 [3.5]
Widowed/divorced				-44 [2.5]	-44 [2.5]
Missing				7 [3.6]	7 [3.6]
**Smoking at the first prenatal visit**					
Non-smoking				Ref.	Ref.
1–9 cig /day				-129 [2.1]	-129 [2.1]
>9 cig/day				-179 [3.5]	-179 [3.5]
Missing				4 [3.4]	4 [3.4]
**Hypertension**					
No				Ref.	Ref.
Yes				-108 [2.5]	-108 [2.5]
**Diabetes**					
No				Ref.	Ref.
Yes				314 [7.1]	314 [7.0]
**Countries economies**					
Low income					-88 [21.1]
Lower-middle income					-52 [17.8]
Upper-middle income					-16 [14.4]
High income (ref)					Ref.

^1^ The table presents measures of association (regression coefficients). Model 1 estimates only the overall mean birthweight of countries. Models 2, 3 and 4 include maternal and new-born characteristics, and Model 5 includes also contextual characteristics. Values in brackets are SE.

**Fig 2:**
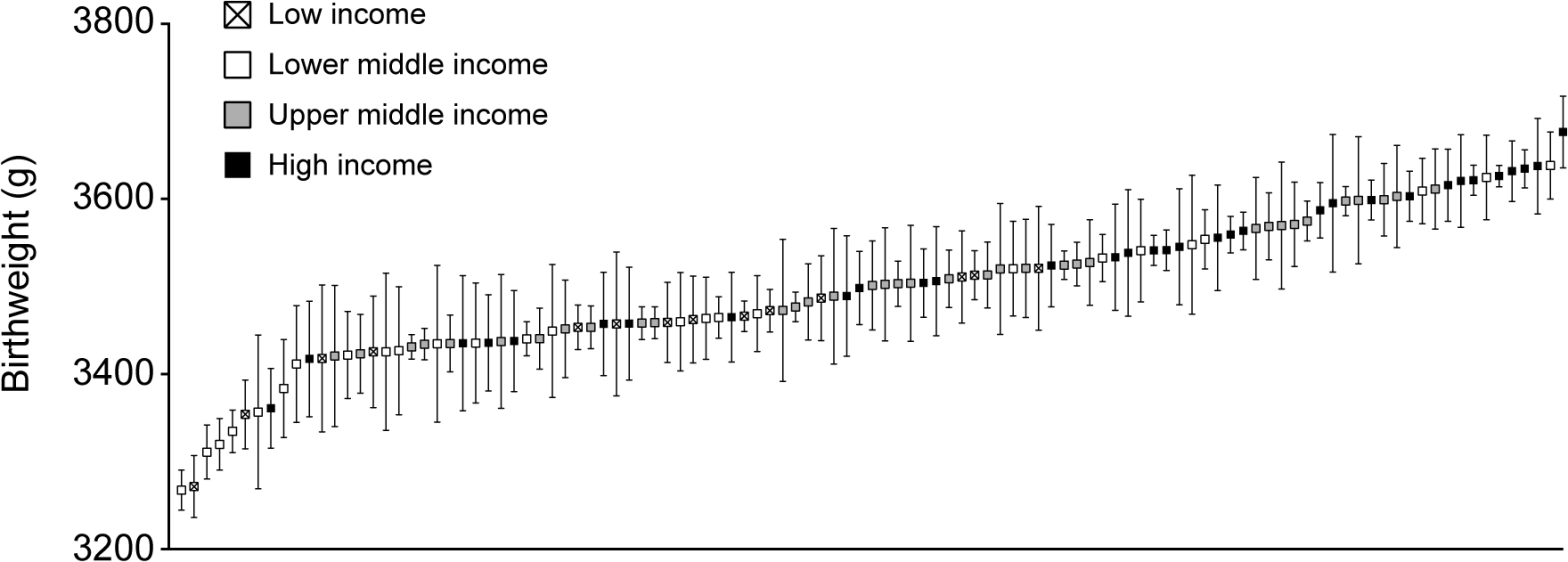
Unadjusted differences in the average birthweight between maternal countries of birth. The values represent the shrunken residuals and their confidence intervals obtained from the multilevel linear regression analysis.

Fig 2 represents the unadjusted ‘league table’ (i.e. Model 1) of MCB ranked according to their mean birthweight. Independently of country economy most countries have a mean birthweight within 3400–3700 g; however nine countries had mean birthwights that were markedly lower (i.e. below 3400 g) than the rest of countries. Two of those countries are in the low income group (Bangladesh, Gambia), six are in the lower-middle income group (India, Pakistan, Sri Lanka, Vietnam, Senegal, Sudan) and one is in the high income group (Japan).

### Measures of variation and general contextual influences

Table 3 provides information about the newborn, mother and MCB components of variance in birthweight. The first model shows that half of the total variance in birthweight is actually at the baby level (i.e. VPC _Newborn_ = 50%). The remaining half is mostly at the maternal level (i.e. VPC _Mother_ = 47% and ICC_Mother_ = 50%), with only 3% of the offspring variance in birthweight being at the MCB level (i.e. VPC/ICC _Mother’s country of birth_ = 3.2%). Addition of mothers’ stature to the model (Model 2) removed 36% of the variance at the MCB level and 6% of the variance at the mother level. Inclusion of the mothers’ age (Model 3) removed an additional 5% of the small residual variance at the MCB level. Addition of more maternal and offspring variables (Model 4) and the MCB variable (Model 5) did not considerably change the VPCs. The intra–MCB correlation of birthweight was slightly higher in Model 4 that included individual information (ICC = 2.5%), than in Models 2 and 3 (ICC = 2.1% and 2.0%, respectively) but this was mainly because of a decrease in variance at the mother and offspring levels due to the inclusion of additional individual-level variables. The inclusion of the WB classification of country economies in the final Model 5 reduced the variance between MCB by an additional 10% (i.e. from 4635 to 3811).

**Table 3:** Multilevel linear regression analysis of babies, mothers and maternal countries of birth, modelling birthweight (in grams)^1^.

	Model 1	Model 2	Model 3	Model 4	Model 5
*Random effects part of the models*					
**Measure of variance (standard error)**					
σ _Mother’s country of birth_	7,699 (1,134)	4,922 (775)	4,520 (711)	4,635 (708)	3,811 (607)
σ _Mother_	111,583 (468)	104,640 (442)	105,398 (468)	85,713 (362)	85,702 (348)
σ _Newborn_	119,871 (350)	120,491 (338)	119,147 (343)	92,740 (269)	92,738 (260)
σ _Total_	239,153	230,053	229,065	183,088	182,251
**Variance partitioning coefficient (VPC) (%)**					
VPC _Mother’s country of birth_	3.2	2.1	2.0	2.5	2.1
VPC _Mother_	46.7	45.5	46.0	46.8	47.0
VPC _Newborn_	50.1	52.4	52.0	50.7	50.9
**Intra-class correlation (ICC) (%)**					
ICC _Mother’s country of birth_	3.2	2.1	2.0	2.5	2.1
ICC _Mother_	49.9	47.6	48.0	49.4	49.1
**Proportional change in variance (PCV) by model (%)**					
PCV _Mother’s country of birth_	Ref.	-36.1	-41.3	-39.8	-50,5
PCV _Mother_	Ref.	-6.2	-5.5	-23.2	-23.2
PCV _Newborn_	Ref.	0.5	-0.6	-22.6	-22.6
PCV _Total_	Ref.	-3.8	-4.2	-23.4	-23.8
Bayesian deviance information Criterion (BDIC)	11790,261	11794,461	11785,356	11894,736	11894,741
Change in BDIC compared with the empty model	Ref.	4,200	4,905	104,475	104,480

^1^ The table presents measures of variance. Model 1 contains only random intercepts at each level and informs on the components of variance across levels. Models 2, 3 and 4 include maternal and newborn characteristics, and Model 5 includes also contextual characteristics (see Table 2 for a list of the variables included in each model). Values in parenthesis are SE.

**Fig 3:**
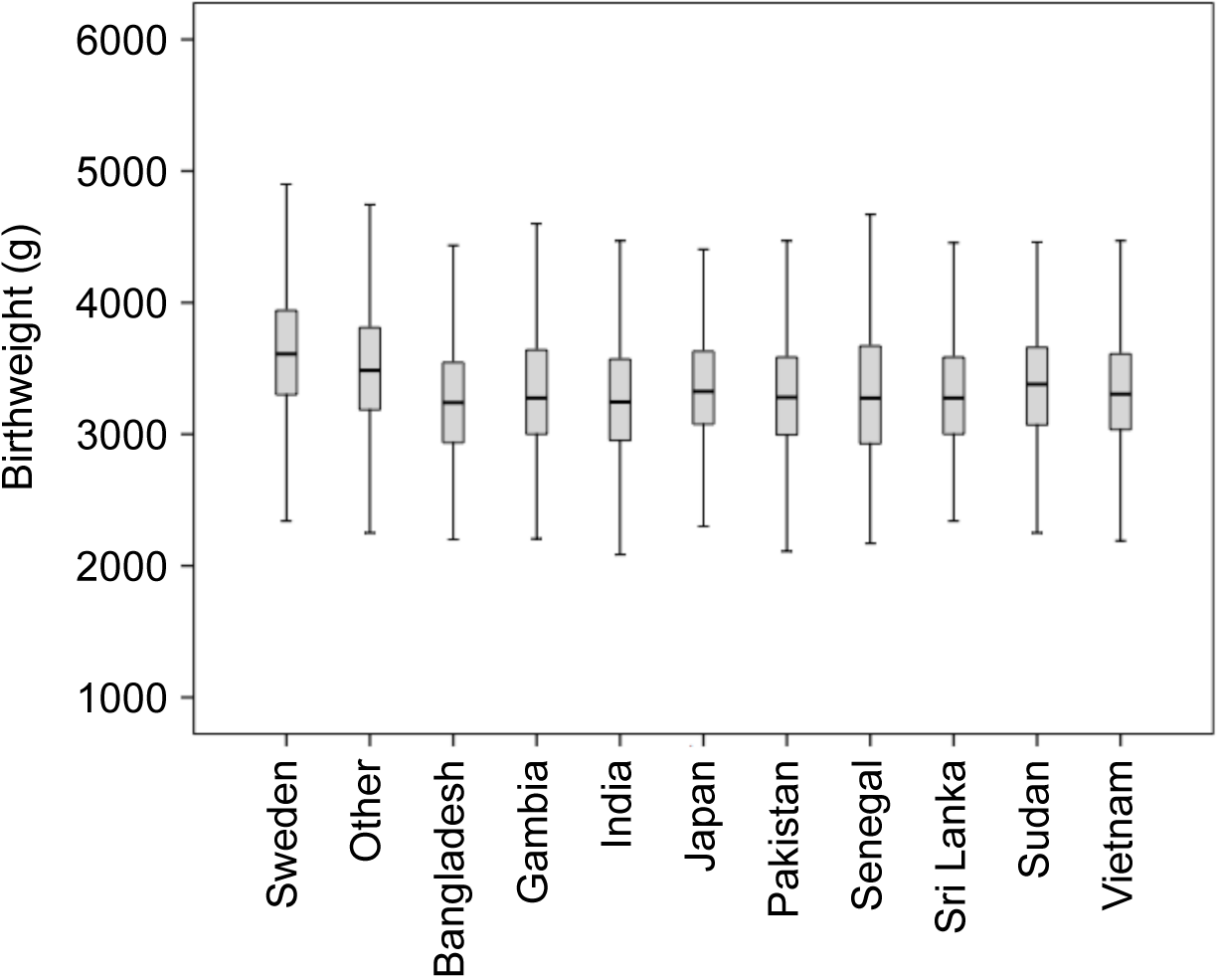
Unadjusted offspring birthweight distributions. Box and whisker plots for mothers born in Sweden (n = 647,953), Bangladesh (n = 905), Gambia (n = 713), India (n = 2636), Japan (n = 503), Pakistan (n = 1273), Senegal (n = 100), Sri Lanka (n = 1357), Sudan (n = 324), and in all other countries (n = 153,219). There is an overlap in distributions corresponding to a low ICC.

Fig 3 represents the crude birthweight distribution within each of the nine MCBs with the lowest mean birthweight, as well as in Sweden and all other countries combined. A simple visual observation of the data suggests the existence of a clear overlap between countries corresponding to a low ICC.

### Sensitivity analyses

In Fig 2 we observed that nine countries had markedly lower mean birthweight than the rest of countries. Excluding those countries from the analysis removed 37% of the variance at the MCB level compared to the ‘empty’ model with all countries included. The intra–MCB correlation in offspring birthweight in the new model was 2.1%.

In Model 2 we found that about a third of the variance at the MCB level was explained by differences in the mothers’ stature, which could suggest that mothers’ stature clusters within countries of birth. To test this hypothesis we used multilevel linear regression to model individual stature as a continuous variable, with mothers at the first level and their countries of birth at the second level. By contrast to birthweight, we found that stature considerably clustered within countries (ICC = 24%).

Finally, we replicated our analysis using data from another dataset that included all births recorded at the Swedish MBR between 1 January 1987 and 31 December 1993. The final sample consisted of 757,811 babies born from 537,093 mothers from 68 different countries of origin. Again only about 3% of the offspring variance in birthweight was at the MCB level (ICC = 2.8%).

## Discussion

In the present study we adopted a multilevel analytical approach and found that the degree of MCB-level clustering in offspring birthweight among women in Sweden was minimal (VPC/ICC = 3.2%). Furthermore, after accounting for individual level variables and socioeconomic circumstances of the country, the ICC became 2.1%. The ICC value can also be interpreted as the size of the correlation in birthweight between two babies randomly sampled from the same MCB. Such a minor ICC for MCB contrasted with the large—expected—clustering of offspring birthweight within mothers (i.e. ICC = 50% in ‘empty’ model), which reflects the strong influence of shared genetic and environmental factors on siblings from the same mother. The low ICC value contrasted moreover with the much higher correlation in stature between mothers with the same country of birth (i.e. ICC = 24%), thus validating our multilevel approach.

The low correlation in birthweight between babies with the same MCB indicates that there is a very high inter-offspring heterogeneity (i.e. within-country variation) around the country average birthweight—a fact that can be visualised in Fig 3. This low clustering suggests that MCB plays a minor role for understanding variation in offspring birthweight in Sweden. That is, knowledge of MCB seems rather irrelevant when it comes to predicting the birthweight of a specific baby and distinguishing its weight from that of another baby, with a different MCB.

Although, to our knowledge, increasing average birthweight in immigrant groups is not an ambition in current public health interventions, our birthweight analysis remains valuable for public health analysts because it exemplifies certain general points using a continuous variable. Thus, conceptually, our findings suggest that a possible public health intervention directed only towards mothers from specific countries (e.g. those with the lowest birthweight averages), as it has been previously suggested [25], would be unjustified since this would convey that (using Rose’s terminology [75]) ‘healthy’ individuals belonging to groups with ‘sick’ average values would unnecessarily be candidates for treatment, while many ‘sick’ individuals would be left outside the intervention because they belong to groups with ‘healthy’ average values.

Our conclusions may still appear counterintuitive. Thus, Fig 2 appears to point to the existence of differences in birthweight between the countries at the extremes of the distribution. In addition, we found a conclusive (i.e. ‘significant’) association between the economic characteristics of the MCB and offspring birthweight. As we discussed elsewhere [41,76], many epidemiologists performing multilevel analyses become confused when they observe a ‘significant’ association between contextual variables and individual health alongside tiny general contextual influences (e.g. VPC close to 0%). This apparent paradox can, however, be unravelled once one recognises that the idea of quantifying general contextual influences by using, for instance, the VPC/ICC, is equivalent to the statistical concept of *discriminatory accuracy* developed in other fields of epidemiology, like the study of risk factors, biomarkers and diagnostic tests [77–79]. It is well recognised that many risk factors and biomarkers are not so useful for predicting individual outcomes, because they have a very low discriminatory accuracy even if they are ‘significantly’ associated with diseases [77].

When facing the evidence of low discriminatory accuracy or low clustering discussed above, many scholars appeal to Rose’s ideas of distinguishing between individual and population levels of analysis and intervention [75,80]. From this perspective there are two kind of causes, *causes of population averages* and *causes of individual cases*, as well as two kind of sicknesses (i.e. sick populations and sick individuals) and two levels of intervention (i.e. public health and clinical medicine). While sympathetic to those concepts, we also propose a multilevel methodological approach that provides a better operationalization of Rose’s ideas [41]. The multilevel analysis allows disentangling of individual from population components of health disparities and provides an efficient instrument for public health analyses. In the multilevel analytical approach the general influence of, as in this case, the MCB is not properly operationalized by measuring differences between country averages. Rather, the general influence of the context is better quantified by measuring the share of the total inter-individual heterogeneity that appears at that specific contextual level [42,50–52], as we have done in our study. We believe that the conceptual multilevel approach we promote [41] is a fundamental, but still not well enough recognised [81], approach for understanding contextual influences on individual health diversity.

Our main point, then, is that measures of association (i.e., differences between group averages) should be complemented by measures of variance and clustering for understanding contextual influences on individual health and for individual risk prediction [41–42]. Needless to say, measures of association may be relevant to questions about average causal effects (ACE); for example, analyses of differences in ethnic group averages might illuminate the causal mechanisms underlying such differences, and may serve as first step in a series of studies and policy discussions regarding reduction of health inequalities. But even for this purpose measures of association should be interpreted with caution. As has been pointed out, the study of variables such as ethnicity, country of birth or race, which are commonly used in social epidemiology, present special difficulties for causal inference [82–84]. In addition, the purpose of the study of ACE [85] is often to understand unobservable individual causal effects (ICE) [86]; yet many described ACE show a very low discriminatory accuracy since individual heterogeneity around averages is high [41]. This situation indicates that in the groups exposed and non-exposed to the risk factor other causal exposures are conditioning the outcome and/or that the effect of the exposure is heterogeneous. Hence knowledge of the discriminatory accuracy of an exposure adds additional information about the causal properties of the exposure beyond that given by measures of association alone. It should be noted that low discriminatory accuracy does not necessarily invalidate a public health intervention because in some instances the medical or social adverse effects of an intervention (e.g. invasion of privacy) are expected to be mild compared to the major public health gains. For example, population wide efforts to reduce smoking are arguably both a medically and socially justifiable strategy to reduce lung cancer even though smoking status is a poor guide to diagnosing lung cancer. However, in many other instances, such as in the case of variables such as ethnicity, country of birth or race, to unnecessarily treat many individuals in the ‘exposed’ group, or leave without treatment many individual in the ‘unexposed’ group is unlikely to be medically and socially justifiable. In sum, the existence of an ACE is a necessary, but not sufficient condition for launching a public health intervention or performing a medical treatment, and we therefore need measures of discriminatory accuracy to help us make appropriate intervention and treatment choices.

### Strength and limitations

It is known that the results of the analyses of variance [87] as well as the estimation of causal effects by the analysis of differences between averages [82] might not be directly extrapolated to other study contexts. Sweden is an established welfare state with a highly developed social protection. These circumstances might attenuate the size of socioeconomic and ethnic disparities in health, including birthweight. It seems necessary to perform similar multilevel analyses in other countries with different welfare and healthcare systems or with other experiences of immigration.

A central issue is the choice of the reference population [88]. In contrast to other studies investigating birthweight, we did not use the native population of mothers as a reference group. Instead, we used the mean value of the overall population of MCB. This strategy might appear inappropriate, because the Sweden-born population of mothers is overrepresented in the analyses. However, we performed sensitivity analyses following different strategies to see whether the results were conditioned by the inclusion of the whole population of mothers. Firstly, we replicated the analysis using a 5% random sample of Swedish-born mothers (meaning 32,398 babies). Secondly, we excluded the whole Swedish-born population from the analysis. In both cases the ICC for MCB remained almost the same (VPC ≈ 3%).

The strength of our analyses is that they are based on a national medical registry covering almost the entire population of residents in Sweden. Because giving birth at home is very unusual in Sweden, nearly all births are registered in the MBR. Also, by including the mother level, our analysis became a quasi-experimental sibling analysis [89–91]. This study design allowed us to account for unknown genetic and environmental variables confounding the association between sibling variables and birthweight. Therefore, concerning individual level associations, our study provides stronger causal evidence than the conventional analyses performed so far. It should be noted, however, that the intra-mother estimations are based on the subpopulation of mothers with two or more children in the database (451,384 babies nested in 211,899 mothers). To investigate the extent to which the results were affected by the inclusion of the maternal level, we performed a sensitivity analysis excluding this level. We observed almost identical results in the random effects analyses (e.g. VPC estimations) and only slight differences in the fixed effect estimations.

On the other hand, it is well known that observational multilevel analysis—like most other observational studies—suffers from problems of exchangeability between the groups being compared, which calls into question the (causal) validity of both general and specific observational contextual measures. This is especially true for characteristics that are originally unchangeable, like maternal country of birth [92–95].

Also, due to data availability, we used the WB’s classification of countries for more recent years, rather than those necessarily corresponding to the time when the immigrant mothers were residing in their countries of origin. In addition, the use of this classification can be questioned, because it is based only on GDP per capita without considering other indicators of social development and economic disparities. We also performed the analysis using the Human Development Index, a composite measure based on indicators of health, education and income that are published by the United Nations [96], but our interpretation of the results did not change.

## Conclusion

Our study suggests that the MCB plays only a minor role in determining individual differences in birthweight, at least in Sweden. Our conclusion is based on the considerable individual heterogeneity around the specific average birthweight values as well as major overlap between countries. Our conclusion not only offers a critique of work in the field of migration, ethnicity and public health that rely heavily on measures of association and information on country of birth, but it also questions numerous investigations in other areas of public health and epidemiology that use population averages to interpret the general contextual influence on health and to propose public health interventions [42,76,97]. Yet, this critique may be *particularly* important to consider for students of country of birth and ethnic differences in health because of the history of racial medicine, the many conceptual and technical problems of research into ethnicity and health, and the perils of ethnic discrimination and stigmatisation. Perhaps a better definition of ‘ethnicity’ as suggested by Stronks and colleagues [37] might increase the value of ethnic categorisations for forecasting birthweight, but the statistical discriminatory accuracy of such a new categorisation for specific outcomes must always be quantified and never be taken at face value. In sum, the multilevel analytical approach we propose allowed us to disentangle population from individual level variance. By doing so, this methodology appears to be a suitable instrument for quantifying the influence of the MCB on offspring birthweight and most likely other health outcomes too.
